# The Role of Prenatal Care and Social Risk Factors in the Relationship between Immigrant Status and Neonatal Morbidity: A Retrospective Cohort Study

**DOI:** 10.1371/journal.pone.0120765

**Published:** 2015-03-27

**Authors:** María Paz-Zulueta, Javier Llorca, Raquel Sarabia-Lavín, Francisco Bolumar, Luis Rioja, Abraham Delgado, Miguel Santibáñez

**Affiliations:** 1 Departamento de Enfermería, Universidad de Cantabria, Santander, Spain; 2 Departamento de Salud Pública, Universidad de Cantabria, Santander, Spain; 3 Instituto de Investigación Marqués de Valdecilla (IDIVAL), Santander, Spain; 4 Centro de Investigación Biomédica en Red (CIBER) de Epidemiología y Salud Pública (CIBERESP), Madrid, Spain; 5 Epidemiology and Biostatistics Program, City University of New York (CUNY) School of Public Health at Hunter College, New York, United States of America; 6 Departamento de Salud Pública, Universidad de Alcalá de Henares, Madrid, Spain; 7 Gerencia de Atención Primaria, Servicio Cántabro de Salud, Santander, Spain; Örebro University, SWEDEN

## Abstract

**Background and Aim:**

Literature evaluating association between neonatal morbidity and immigrant status presents contradictory results. Poorer compliance with prenatal care and greater social risk factors among immigrants could play roles as major confounding variables, thus explaining contradictions. We examined whether prenatal care and social risk factors are confounding variables in the relationship between immigrant status and neonatal morbidity.

**Methods:**

Retrospective cohort study: 231 pregnant African immigrant women were recruited from 2007–2010 in northern Spain. A Spanish population sample was obtained by simple random sampling at 1:3 ratio. Immigrant status (Spanish, Sub-Saharan and Northern African), prenatal care (Kessner Index adequate, intermediate or inadequate), and social risk factors were treated as independent variables. Low birth weight (LBW < 2500 grams) and preterm birth (< 37 weeks) were collected as neonatal morbidity variables. Crude and adjusted odds ratios (OR) were estimated by unconditional logistic regression with 95% confidence intervals (95% CI).

**Results:**

Positive associations between immigrant women and higher risk of neonatal morbidity were obtained. Crude OR for preterm births in Northern Africans with respect to nonimmigrants was 2.28 (95% CI: 1.04–5.00), and crude OR for LBW was 1.77 (95% CI: 0.74–4.22). However, after adjusting for prenatal care and social risk factors, associations became protective: adjusted OR for preterm birth = 0.42 (95% CI: 0.14–1.32); LBW = 0.48 (95% CI: 0.15–1.52). Poor compliance with prenatal care was the main independent risk factor associated with both preterm birth (adjusted OR inadequate care = 17.05; 95% CI: 3.92–74.24) and LBW (adjusted OR inadequate care = 6.25; 95% CI: 1.28–30.46). Social risk was an important independent risk factor associated with LBW (adjusted OR = 5.42; 95% CI: 1.58–18.62).

**Conclusions:**

Prenatal care and social risk factors were major confounding variables in the relationship between immigrant status and neonatal morbidity.

## Introduction

Available literature evaluating the association between neonatal morbidity and immigrant status presents contradictory results. Some studies show risks that are similar to or lower than those for nonimmigrant women. These are related to what has been called the “healthy immigrant effect” or the “epidemiologic paradox”, which states that immigrant women are likely to have better health outcomes, including reproductive outcomes, because they are younger and have healthier lifestyles. This effect was first described in Latino immigrants (mainly Mexican women) in the United States [1] and has also been described in African immigrant women in Europe [2–4] and in Spain [5–7].

In contrast, other studies identify higher risks for preterm birth or low birth weight among pregnant immigrant women [8,9]. However, these studies have not taken into account poorer compliance with prenatal care in this population as a confounding variable. Greater risk of neonatal morbidity (very preterm birth and very low birth weight) in the immigrant population was reduced after adjustment for prenatal care in a recently published cross-sectional study conducted in Spain, suggesting that compliance with prenatal care could play an important role as a confounding variable [10].

In addition, several studies have shown that immigrant women from poor countries live under worse conditions and in worse socioeconomic situations in their host countries, and have a greater risk of inequality in the social determinants of health when compared with nonimmigrant women. It places them in positions of social risk and vulnerability [11–14]. Social risk has also been associated with poorer neonatal outcomes [15–17], and could thus be another important confounding variable in the relationship between immigrant status and neonatal morbidity.

According to this rationale, we hypothesized that the association between immigrant status and a higher risk of neonatal morbidity could be spurious owing to confounding caused by social risk factors or poorer prenatal care, both of which are associated simultaneously with immigrant status and neonatal morbidity. This could explain the contradictory findings in published studies.

Feminization of the migration flows to Spain has developed over the last decade, and the increase in foreign mothers giving birth in Spain has been studied in various fields [17,18]. It is estimated that more than half of female immigrants are of reproductive age, which means that many of their social and health needs have to do with issues of reproduction and maternity [19,20]. In 2010, the majority of foreign women who gave birth in Spain came from Africa, and were mainly from Morocco [21].

We undertook a retrospective cohort study to examine whether the lack of adequate prenatal care and the existence of social risk factors are confounding variables in the relationship between the status of African immigrant women (AIW) and neonatal morbidity.

## Materials and Methods

Approval of the research protocol was obtained from the Clinical Research Ethics Committee of Cantabria before data acquisition began on October 8, 2010. Patient records and information were anonymized and de-identified prior to analysis.

### Study design and participants

In this retrospective cohort study, the study population comprised AIW with uncomplicated pregnancies and delivery dates between January 1, 2007 and December 31, 2010, who attended one of the 41 primary health care centers in the Cantabria region of northern Spain. In the Spanish National Health System, prenatal care for an uncomplicated pregnancy (a pregnancy with no established maternal or obstetric factors that could increase the risk for maternal or fetal morbidity) is carried out by a primary health care midwife and a general practitioner.

We undertook a search using the clinical databases of the Cantabrian primary health care centers. Patient country of origin was subsequently collated with the Connectis Consulting Services Civitas Population Information System. We found 264 births in 237 pregnant AIW during the study period. Among these births, both first and second pregnancies were identified. Of the 264 births, 27 were second pregnancies. We included only information about first pregnancies in our analysis. In the case of six pregnant women, follow-up was referred to an obstetrician owing to a change in their status from uncomplicated to complicated pregnancy. Because these women were not fully under the care of a primary health care center, the final study population was reduced to 231 pregnant AIW with uncomplicated pregnancies. A flow diagram showing the final included AIW study population and their country of origin can be found in S1 Fig.

A Spanish population sample was obtained by simple random sampling using a 1:3 ratio, stratified by the primary health care centers of the 231 AIW study participants. Thus, we defined a population of 693 pregnant Spanish women. Of these 693 Spanish women, 66 were excluded because their status changed to complicated pregnancy and they were referred to an obstetrician. Therefore, the final population for the comparative analysis was 627 Spanish women with uncomplicated pregnancies. A flow diagram showing the final population of Spanish women included in the study is provided in S2 Fig.

### Data sources and variables

#### Measures

Information for each pregnant woman included in our study was obtained from the computerized clinical databases of each primary health care center and from hospital birth records.

The adequacy of prenatal care was estimated based on the Kessner Index (KI) [22]. The KI combines three variables: the start of prenatal care, the total number of consultations, and the length of pregnancy. The KI makes an adjustment to the number of consultations considered adequate for pregnancies that finish before the predicted birth date. In the case of a terminated pregnancy, prenatal care is considered adequate if the first prenatal visit took place before week 14 and at least nine visits were made. Prenatal care is considered inadequate if the first visit was after week 28 or fewer than four prenatal visits were made. With cases that did not fall into either of these two categories, prenatal care is considered intermediate.

In addition, we created our own index (OI) based on seven quality indicators found in current national and international guidelines [23–25]: (1) prenatal care before week 12; (2) folic acid supplementation before week 8; (3) ultrasound scan between 11 and 14 weeks of amenorrhea; (4) ultrasound screening for fetal abnormalities between weeks 18 and 20; (5) hepatitis B and HIV screening in the first trimester; (6) gestational diabetes screening between weeks 24 and 28; and (7) at least six completed prenatal visits. Prenatal care was considered adequate based on compliance with all OI indicators. Care was considered intermediate if one to three indicators were unmet and inadequate if four or more indicators were unmet.

The current Spanish prenatal care protocol sets the minimum periodicity of visits at one per month (at weeks 6, 10, 14, 20, 24, 28, 32, and 36) until week 36 and once every two weeks between weeks 36 and 40. Therefore, by week 28, any pregnant woman having received adequate prenatal care should have completed six prenatal visits and undergone the appropriate checks in compliance with all the quality indicators.

The criteria identifying the need to refer pregnant women to a social worker (SW) are based on certain characteristics that increase their social risk and place them in a position of vulnerability or chronic stress. These characteristics include precarious living conditions, such as insufficient income (having no income support or partner is unemployed), living in a small or unhealthy environment, having an inadequate diet, and potential situations of abuse or domestic violence. In the official Spanish prenatal care protocol, when the midwife or general practitioner identifies any social risk factor, the pregnant woman affected is then referred to an SW, with a note made in the protocol regarding the necessity of referral to an SW.

The main neonatal morbidity variables considered were low weight (less than 2,500 grams at birth) and preterm birth (less than 37 weeks).

#### Statistical analyses

Discrete variables were expressed as counts (percentage) and continuous variables as mean (± standard deviation). Statistical differences between groups were assessed with the chi-squared test using the Yates correction or Fisher’s exact test, when appropriate, for categorical variables. The Student’s *t*-test was used for continuous variables. Immigrant status of the study participants was categorized as Spanish, Sub-Saharan African or Northern African. The Sub-Saharan and Northern African categories were then combined to create a dichotomous variable “Spanish” versus “African”. The adequacy of prenatal care, as measured by the KI and OI indexes, was categorized as adequate, intermediate, or inadequate.

Preterm birth (< 37 weeks) and low birth weight (< 2500 grams) were treated as dichotomous dependent variables in the regression models. For low birth weight, the normal weight category (2500–3999 grams) was treated as the reference category, and macrosomic birth weights (≥ 4000 grams) were excluded from the analysis.

A basic unconditional regression model was used, adding only the frequency-matching variable; the primary health care center. Odds ratios (ORs) with 95% confidence intervals (95% CI) estimated by this basic model were treated as crude odds ratios (ORc). To analyze the effect of social risk factors and compliance with prenatal care as confounding variables in the relationship between immigrant status and neonatal morbidity, these variables were first added to the basic logistics model separately, and then both were added together. Last, analysis using an overall regression model was performed, adjusting for immigrant status, referral to a social worker (yes/no), prenatal care, age of the woman (as a continuous variable), and number of previous pregnancies (as a continuous variable).

Tests for OR trends were calculated for the ordinal KI and OI index categories using logistic models that included categorical terms as continuous variables. For these trend tests, we used a likelihood ratio test. The alpha error was set at 0.05, and all *p*-values were bilateral. All statistical analyses were conducted using IBM SPSS Statistics version 21.0.

## Results

The majority of AIW came from countries in the north of Africa: n = 160 (69.3%). Participants were mainly from Morocco: n = 144 (62.3%), followed by Sub-Saharan countries such as Nigeria: n = 18 (7.8%), or Senegal: n = 18 (7.8%) and Cameroon: n = 17 (7.4%). Table 1 shows characteristics of the mothers and their newborns, as well as the type of birth and prenatal care, according to country of origin.

**Table 1:** Characteristics of the mothers and their newborn, depending on their country of origin. Cantabria (Spain): African immigrants and Spanish women 2007–2010.

	Spanish (N = 627)		North African (N = 160)		Moroccan (N = 144)		Sub-Saharan (N = 71)	
	n	(%)	n	(%)	n	(%)	n	(%)
Maternal age: Mean [SD]^a^	31.3 [4.1]		27.7 [6.0]***		27.6 [6.0]***		28.3 [5.2]***	
≤ 16	0	(0)	1	(0.6)	1	(0.7)	0	(0)
17–34	490	(78.1)	135	(84.4)	124	(86.1)	62	(87.3)
≥ 35	137	(21.9)	24	(15.0)	19	(13.2)	9	(12.7)
N° previous pregnancies: Mean [SD]^a^	0.53 [0.815]	n = 627	0.83 [1.074]	n = 159	0.82 [1.111]	n = 143	0.97 [1.035]	n = 70
Smokers	100	(16.5)^b^	0	(0)***	0	(0)***	1	(1.4)**
Missing values	21	(3.3)	0	(0)	0	(0)	0	(0)
Alcohol	6	(1.0)^b^	0	(0)	0	(0)	0	(0)
Missing values	21	(3.3)	0	(0)	0	(0)	0	(0)
Speak Spanish^c^								
Missing values	—	—	12	(7.5)	12	(8.3)	4	(5.6)
Sufficient	—	—	21	(14.2)^b^	20	(15.2)^b^	14	(20.9)^b^
Insufficient	—	—	127	(85.8)^b^	112	(84.8)^b^	53	(79.1)^b^
Read Spanish^d^								
Missing values	—	—	13	(8.1)	13	(9.0)	4	(5.6)
Sufficient	—	—	9	(6.1)^b^	9	(6.9)^b^	2	(2.9)^b^
Insufficient	—	—	138	(93.9)^b^	122	(93.1)^b^	65	(97.1)^b^
Write Spanish^e^								
Missing values	—	—	13	(8.1)	13	(9.0)	4	(5.6)
Sufficient	—	—	9	(6.1)^b^	9	(6.9)^b^	2	(2.9)^b^
Insufficient	—	—	138	(93.9)^b^	122	(93.1)^b^	65	(97.1)^b^
Referral SW^f^								
Missing values	0	(0)	3	(1.9)	3	(2.1)	2	(2.8)
No referral	596	(95.1)	88	(56.1)^b^	81	(57.4)^b^	31	(44.9)^b^
Referral	31	(4.9)	69	(43.9)*** ^b^	60	(42.6)*** ^b^	38	(55.1)*** ^b^
Kessner Index (KI)^g^								
Missing values	0	(0)	0	(0)	0	(0)	0	(0)
Adequate	476	(75.9)	34	(21.3)***	33	(22.9)***	16	(22.5)***
Intermediate	144	(23.0)	80	(50.0)***	73	(50.7)***	36	(50.7)***
Inadequate	7	(1.1)	46	(28.7)***	38	(26.4)***	19	(26.8)***
Own Index (OI)^h^								
Missing values	0	(0)	1	(0.6)	1	(0.7)	2	(2.8)
Adequate	491	(78.3)	41	(25.8)*** ^b^	40	(27.9)*** ^b^	21	(30.4)*** ^b^
Intermediate	129	(20.6)	68	(42.8)*** ^b^	61	(42.7)*** ^b^	31	(44.9)*** ^b^
Inadequate	7	(1.1)	50	(31.4)*** ^b^	42	(29.4)*** ^b^	17	(24.7)*** ^b^
Fetal age, weeks								
Preterm Birth (<37)	21	(3.3)	11	(6.9)*	9	(6.3)	3	(4.2)
Full term neonate (≥37)	606	(96.7)	149	(93.1)	135	(93.7)	68	(95.8)
Birth weight, grams								
Low weight (<2500)	21	(3.3)	8	(5.0)	7	(4.9)	3	(4.2)
Normal weight (2500–3999)	569	(90.8)	135	(84.4)	122	(84.7)	60	(84.5)
Macrosomic (≥4000)	37	(5.9)	17	(10.6)*	15	(10.4)	8	(11.3)

^a^ Standard Deviation.

^b^ % Valid when missing.

^c^ Insufficient Oral Spanish language skill.

^d^ Insufficient Reading Spanish skills.

^e^ Insufficient Writing Spanish skills.

^f^ Referral to social worker because of Social Risk factor detection.

^g^ Prenatal care as assessed by KI: Adequate: first consultation before week 14 and at least 9 consultations during gestation. Inadequate: start of care after week 28, or less than 4 consultations during gestation. Intermediate: the other combinations.

^h^ Prenatal care as assessed by OI: Adequate: all 7 indicators of the Spanish Health Service pregnancy care protocol met. Inadequate: ≥4 indicators unmet. Intermediate: 1–3 indicators unmet.

*p ≥ 0.01 - < 0.05

** p ≥ 0.001 - < 0.01

*** p < 0.001

The AIW were on average 3.45 years younger than Spanish women (95% CI: 2.75–4.15, *p*<0.001) and showed healthier lifestyles with respect to alcohol consumption and smoking. A total of 47% of AIW were referred to an SW by midwives owing to detection of social risk, compared with 5% of Spanish women (ORc 17.29; 95% CI:11.07–26.99, *p*<0.001). Prenatal care was considered to be adequate in 27% and 22% of AIW using the KI and OI, respectively, compared with 78% and 76% of Spanish women (*p*<0.001).

The AIW had a greater percentage of preterm births and low birth weight cases.

Tables 2 and 3 show the crude and adjusted associations between immigrant status and the risk of preterm birth and low birth weight, respectively. All AIW, especially those from Northern Africa, were at higher risk of preterm birth (ORc 2.28; 95% CI:1.04–5.00). However, these associations were spurious, becoming protective after adjusting for compliance with prenatal care and social risk factors (adjusted OR for Northern Africans 0.42; 95% CI: 0.14–1.32). This association did not change after including maternal age and number of previous pregnancies in the models (adjusted OR Northern Africans 0.45; 95% CI: 0.14–1.44).

**Table 2:** Crude and adjusted associations between the immigrant status, other predefined variables (social risk factors and compliance with prenatal care), and the risk of neonatal morbidity (preterm birth).

	PRETERM BIRTH^a^									
	ORc^b^	(95%CI)	ORa1^c^	(95%CI)	ORa2^d^	(95%CI)	ORa3^e^	(95%CI)	ORa4^f^	(95%CI)
African ^g^	1.92	(0.95–3.89)	0.91	(0.37–2.26)	0.42	(0.15–1.18)	0.36	(0.12–1.06)	0.38	(0.13–1.14)
Sub-saharan	1.20	(0.32–4.46)	0.53	(0.12–2.28)	0.27	(0.06–1.24)	0.23	(0.05–1.10)	0.23	(0.05–1.17)
North-African	2.28	(1.04–5.00)	1.10	(0.42–2.85)	0.50	(0.17–1.49)	0.42	(0.14–1.32)	0.45	(0.14–1.44)
Social risk factors ^h^	4.05	(1.95–8.39)	4.38	(1.75–10.94)	—		2.02	(0.71–5.77)	2.28	(0.76–6.87)
Kessner Index (KI) ^i^										
KI intermediate	3.32	(1.44–7.68)	—		4.66	(1.87–11.59)	3.97	(1.54–10.24)	4.02	(1.55–10.45)
KI inadequate	11.37	(4.26–30.30)	—		24.12	(6.23–93.35)	16.46	(3.81–71.06)	17.05	(3.92–74.24)
*linear p trend*	*< 0*.*001*				*< 0*.*001*		*< 0*.*001*		*< 0*.*001*	
Own Index (OI) ^j^										
OI intermediate	1.34	(0.57–3.12)	—		1.45	(0.59–3.57)	1.21	(0.48–3.08)	1.23	(0.48–3.13)
OI inadequate	5.54	(2.30–13.34)	—		6.55	(1.88–22.86)	3.92	(1.05–14.60)	4.08	(1.08–15.36)
*linear p trend*	*0*.*001*				*0*.*007*		*0*.*069*		*0*.*064*	

Cantabria (Spain): African immigrants and Spanish women 2007–2010.

^a^ Newborn less than 37 weeks at birth.

^b^ Odds Ratio and 95% Confidence Intervals. ORc denotes “crude Odds ratio” = the basic regression model adding only the frequency matching variable: Primary Health Center (PHC).

^c^ ORa1 denotes “adjusted” OR adding to the basic model: immigrant status and social risk factors.

^d^ ORa2 denotes “adjusted” OR adding to the basic model: immigrant status and prenatal care.

^e^ ORa3 denotes “adjusted” OR adding to the basic model: immigrant status, social risk factors, and prenatal care.

^f^ ORa4 denotes “adjusted” OR adding to the basic model: immigrant status, social risk factors, prenatal care, maternal age at delivery (continuous), and number of previous pregnancies (continuous).

^g^ African immigran women (Sub-Saharan and North-African combined)

^h^ Referral to a social worker because of social risk factor detection.

^i^ Prenatal care as assessed by KI: Adequate: first consultation before week 14 and at least 9 consultations during gestation. Inadequate: start of care after week 28, or less than 4 consultations during gestation. Intermediate: the other combinations.

^j^ Prenatal care as assessed by OI: Adequate: all 7 indicators of the Spanish Health Service pregnancy care protocol met. Inadequate: ≥4 indicators unmet. Intermediate: 1–3 indicators unmet.

**Table 3:** Crude and adjusted associations between the immigrant status, other predefined variables (social risk factors and compliance with prenatal care), and the risk of neonatal morbidity (low weight). Cantabria (Spain): African immigrants and Spanish women 2007–2010.

	LOW WEIGHT^a^									
	ORc^b^	(95%CI)	ORa1^c^	(95%CI)	ORa2^d^	(95%CI)	ORa3^e^	(95%CI)	ORa4^f^	(95%CI)
African ^g^	1.50	(0.70–3.21)	0.62	(0.22–1.70)	0.50	(0.19–1.35)	0.36	(0.12–1.09)	0.39	(0.12–1.30)
Sub-saharan	1.05	(0.29–3.89)	0.28	(0.05–1.54)	0.36	(0.08–1.57)	0.18	(0.03–1.02)	0.22	(0.03–1.36)
North-African	1.77	(0.74–4.22)	0.82	(0.28–2.38)	0.58	(0.20–1.73)	0.48	(0.15–1.52)	0.48	(0.14–1.68)
Social risk factors ^h^	3.27	(1.49–7.16)	4.51	(1.67–12.18)	—		2.83	(0.96–8.33)	5.42	(1.58–18.62)
Kessner Index (KI)^i^										
KI intermediate	3.19	(1.38–7.36)	—		4.26	(1.70–10.70)	3.28	(1.26–8.51)	3.51	(1.31–9.43)
KI inadequate	5.05	(1.77–14.44)	—		8.93	(2.32–34.44)	4.41	(1.01–19.22)	6.25	(1.28–30.46)
*linear p trend*	*0*.*001*				*0*.*001*		*0*.*022*		*0*.*011*	
Own Index (OI)^j^										
OI intermediate	1.12	(0.47–2.67)	—		1.05	(0.41–2.70)	0.82	(0.31–2.18)	1.01	(0.37–2.74)
OI inadequate	2.35	(0.86–6.42)	—		2.00	(0.57–6.97)	0.83	(0.21–3.31)	1.25	(0.29–5.45)
*linear p trend*	*0*.*145*				*0*.*360*		*0*.*733*		*0*.*809*	

^a^ Newborn less than 2,500 grams at birth

^b^ Odds Ratio and 95% Confidence Intervals. ORc denotes “crude Odds ratio” = the basic regression model adding only the frequency matching variable: Primary Health Center (PHC).

^c^ ORa1 denotes “adjusted” OR adding to the basic model: immigrant status and social risk factors.

^d^ ORa2 denotes “adjusted” OR adding to the basic model: immigrant status and prenatal care.

^e^ ORa3 denotes “adjusted” OR adding to the basic model: immigrant status, social risk factors, and prenatal care.

^f^ ORa4 denotes “adjusted” OR adding to the basic model: immigrant status, social risk factors, prenatal care, maternal age at delivery (continuous), and number of previous pregnancies (continuous).

^g^ African immigran women (Sub-Saharan and North-African combined)

^h^ Referral to a social worker because of social risk factor detection.

^i^ Prenatal care as assessed by KI: Adequate: first consultation before week 14 and at least 9 consultations during gestation. Inadequate: start of care after week 28, or less than 4 consultations during gestation. Intermediate: the other combinations.

^j^ Prenatal care as assessed by OI: Adequate: all 7 indicators of the Spanish Health Service pregnancy care protocol met. Inadequate: ≥4 indicators unmet. Intermediate: 1–3 indicators unmet.

A lack of compliance with prenatal care based on KI was the main independent risk factor for preterm birth. A very significant dose–response pattern (*p*-trend<0.001) was found. The greater the lack of prenatal care compliance, the greater the association for preterm birth (adjusted OR for intermediate care 4.02; 95% CI: 1.55–10.45; adjusted OR for inadequate care 17.05; 95% CI: 3.92–74.24). Existence of social risk factors was an independent risk factor for preterm birth (ORc 4.05; 95% CI:1.95–8.39), but this association diminished in the overall adjusted model (adjusted OR 2.28; 95% CI: 0.76–6.87).

With respect to low birth weight, crude associations between status as an immigrant and low birth weight changed, becoming protective after adjusting for poorer compliance with prenatal care and/or social risk factors. Lack of compliance with prenatal care based on the KI was also an independent risk factor for low birth weight, with a very significant dose–response pattern (*p*-trend = 0.011) found (adjusted OR intermediate care 3.51; 95% CI:1.31–9.43; adjusted OR for inadequate care 6.25; 95% CI: 1.28–30.46). Existence of social risk factors was an important independent risk factor associated with low birth weight (adjusted OR 5.42; 95% CI: 1.58–18.62).

## Discussion

Poor compliance with prenatal care was the main risk factor associated with neonatal morbidity in our study, with statistically significant dose–response patterns found. The existence of social risk factors was independently associated with neonatal morbidity, especially low birth weight. Here, both variables played an important role as confounding variables, being simultaneously associated with immigrant status and neonatal morbidity, and causing a spurious relationship between immigrant status and higher risk of neonatal morbidity.

As mentioned above, crude associations between status as an AIW and neonatal morbidity, which are supported by different studies in Spain [17], Europe [20,26–28], and the United States [29,30], were not maintained in the multivariable models that included poor compliance with prenatal care or the existence of social risk factors. This supports the results of Castelló et al. [10], in which associations between being an immigrant and the risk of very preterm birth and very low birth weight were weaker when adjusting for adequacy of prenatal care.

The meta-analysis published by Bollini et al. [31] indirectly supports our results. In 12 countries receiving immigrants that were studied from 1966 to 2004, pregnant immigrant women showed a greater risk of low birth weight and preterm delivery. These risks were clearly and significantly reduced in those countries with stronger integration policies that lowered both access barriers to prenatal care and the existence of social risk factors, even after adjusting for maternal age at the time of birth.

After adjustment, status as an immigrant was protective in our study. The AIW in our study were younger, but also had healthier lifestyles in terms of alcohol use and smoking. This could explain the adjusted protective effect obtained in the multivariable regression model that also included maternal age as a covariate. This would support the “healthy immigrant” effect or “epidemiologic paradox”, which state that immigrant women have better reproductive outcomes because they are younger and lead healthier lifestyles in comparison with nonimmigrant women.

The greater associations found here between poorer compliance with prenatal care and neonatal morbidity are supported by several studies, specifically in immigrant populations [29,32–34]. The existence of a dose-response pattern would provide additional support for a real association, and not owing to chance.

The KI is a classical prenatal care indicator. This index, similar to other published indexes such as the Adequacy of Prenatal Care Utilization (APNCU), only considers the number of visits during pregnancy but not their content. The APNCU index considers the same three parameters considered in the KI index (gestational week at first prenatal care visit, number of prenatal visits, and duration of pregnancy), but in a different way [35]. In Spain, Delgado-Rodriguez et al. compared the KI with the APNCU index to examine the degree of association of both indexes with respect to risk of preterm birth [35]. The KI was considered a better predictor for preterm birth than the APNCU. Therefore, we chose the former for our work.

The latest recommendations from international organizations, such as the World Health Organization or National Institute for Health and Care Excellence [24,25], place more importance on other prenatal care quality indicators than on the number of visits, with at least six visits considered optimal. This arises from the fact that no statistically significant differences have been found with respect to neonatal and maternal morbidity when more than six visits are completed, as long as the other quality indicators are complied with (i.e., first visit before week 12, folic acid supplementation before week 8, and corresponding blood tests and screenings) [36,37].

On the basis of these recommendations, in addition to the KI, we used the OI in this study, which considers both the number of antenatal care visits and compliance with the other quality indicators. According to the OI, six visits for a term pregnancy would be considered adequate prenatal care, as long as the remaining prenatal care quality indicators are complied with.

However, according to the KI, pregnant women who have complied with the remainder of the Spanish prenatal care quality indicators, but who have only completed six, seven or eight visits, would be considered to have had intermediate prenatal care by virtue of having completed fewer than nine prenatal visits.

According to the OI, 12 out of 62 AIW and 15 out of 491 Spanish women were classified in the adequate care category; however, these women were classified as intermediate care by the KI, simply because they had six to eight prenatal visits despite having complied with the remaining quality indicators. Among these 12 AIW, there was one case of low birth weight, and among the 15 Spanish women, there were five cases of preterm birth, and six cases of low birth weight. These extra cases of preterm births and low birth weight newborns in the adequate category according to the OI would explain the lower associations found using the OI when compared with the KI, both in AIW as well as in Spanish women.

Therefore, our results do not support those of Villar et al. [36,37], because we found a greater prevalence of neonatal morbidity in pregnant women who complied with the quality indicators proposed by those authors and recommended by the World Health Organization (including at least six completed prenatal visits), but who had fewer than nine prenatal visits, which is the minimum number of visits used in the Kessner Index.

Although the relationship between social risk factors and risk of neonatal morbidity has been less investigated, our results are supported by several studies. Sparks et al. (2009) showed that preterm birth differences in the United States were more associated with social risk factors than with country of origin [16]. Garcia-Subirats et al. showed that the unemployment rate was associated with preterm births in every maternal age group [17]. An association between low birth weight and social risk factors, such as being a teenage mother or single mother, is also supported [15]. A relationship between the existence of social risk factors and maternal stress biomarkers, together with a greater rate of genital tract infections, could explain the pathogenic basis of this association [38–40].

In retrospective studies based on secondary information (records), one of the main limitations could be the low quality of the information, owing either to incomplete records or a lack of agreement among the different records. In our study, information about the main variables was collected in more than 97% of women, and agreement for the country of origin was 100%. Another caveat relates to the lack of availability of other potential risk factors, which are unavailable from the secondary registers used in our study.

In conclusion, poor compliance with prenatal care and the existence of social risk factors were major confounding variables in the relationship between immigrant status and neonatal morbidity in our study. Further epidemiological studies should include both variables as confounding variables to prevent biased results.

Regarding impact in the clinical setting, our results have identified poor compliance with prenatal care as the main independent risk factor associated with both preterm birth and low birth weight, in both immigrant and nonimmigrant pregnant women, which supports the importance of compliance with more than six prenatal visits. The existence of social risk factors was an important independent risk factor associated with low birth weight. This lends support for the joint role played by social workers and midwives in maximizing the level of proper prenatal care among pregnant women with increased social risk.

## Supporting Information

S1 Fig Flow diagram showing African Immigrant Women finally included and their country of origin. *An uncomplicated pregnancy was defined as a pregnancy without established maternal or obstetric risk factors that could increase the risk for maternal or fetal morbidity, and carried out by a primary health-care midwife and a general practitioner in the primary health-care centers.
(TIF)

S2 Fig Flow diagram showing inclusion & exclusion criteria, for the Spanish women obtained by simple random sampling using a 1:3 ratio. *An uncomplicated pregnancy was defined as a pregnancy without established maternal or obstetric risk factors that could increase the risk for maternal or fetal morbidity, and carried out by a primary health-care midwife and a general practitioner in the primary health-care centers.
(TIF)
