# Supplementary figures and images for: The Role of Prenatal Care and Social Risk Factors in the Relationship between Immigrant Status and Neonatal Morbidity: A Retrospective Cohort Study
Source: PLoS One. 2015 Mar 27;10(3):e0120765. doi: 10.1371/journal.pone.0120765 (PMC4376771; doi:10.1371/journal.pone.0120765)

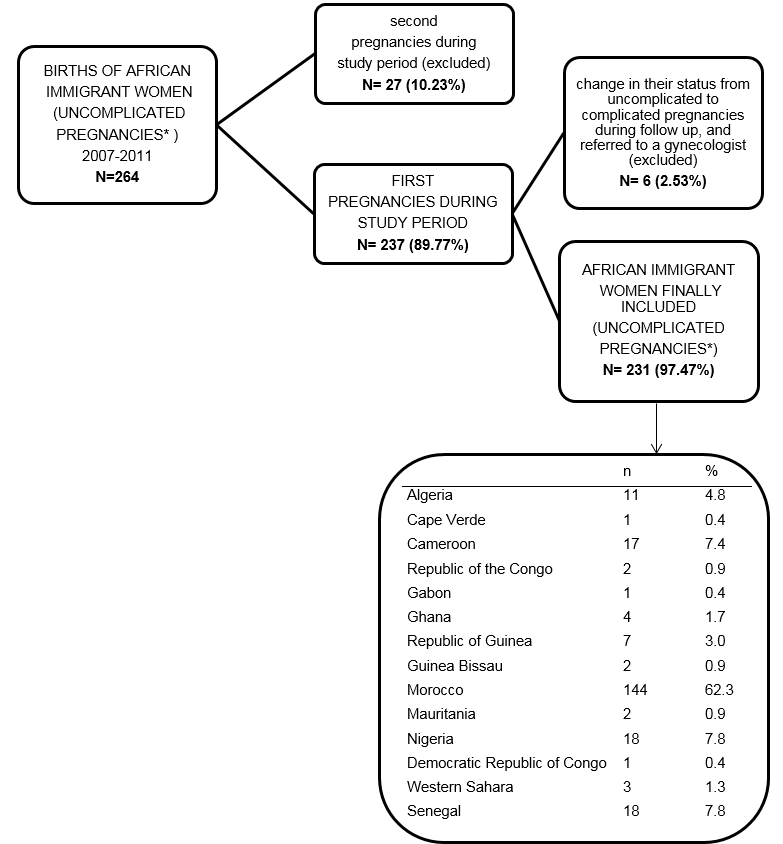

Supplement: S1 Fig — *An uncomplicated pregnancy was defined as a pregnancy without established maternal or obstetric risk factors that could increase the risk for maternal or fetal morbidity, and carried out by a primary health-care midwife and a general practitioner in the primary health-care centers. (TIF) [file pone.0120765.s001.tif]

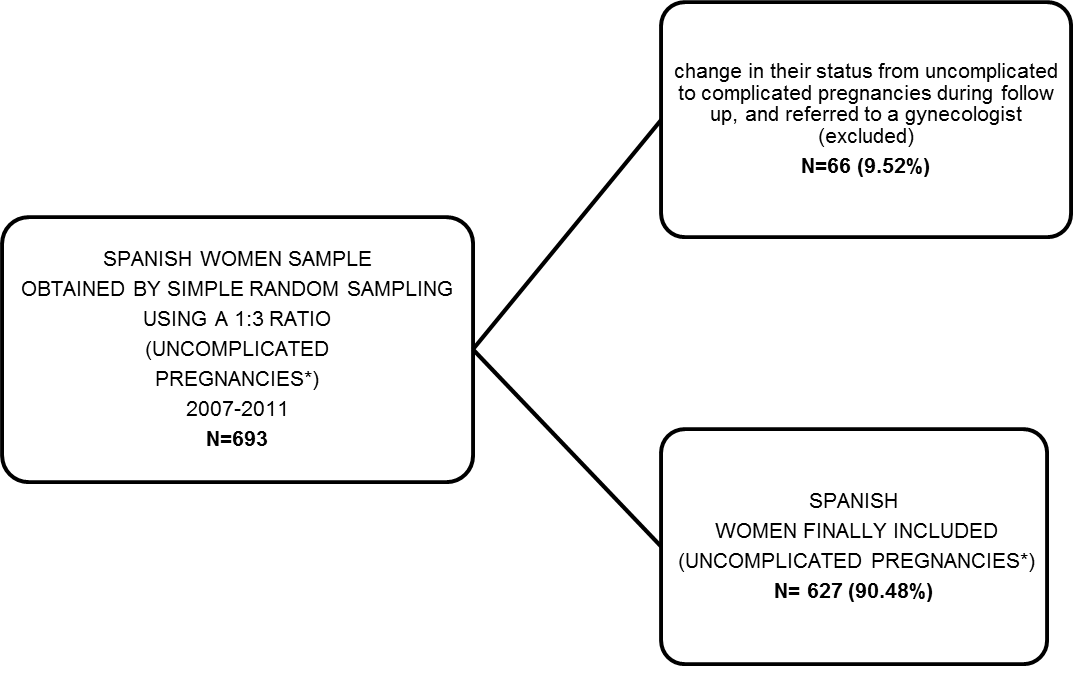

Supplement: S2 Fig — *An uncomplicated pregnancy was defined as a pregnancy without established maternal or obstetric risk factors that could increase the risk for maternal or fetal morbidity, and carried out by a primary health-care midwife and a general practitioner in the primary health-care centers. (TIF) [file pone.0120765.s002.tif]
